# High thermoelectric performance of flexible nanocomposite films based on Bi_2_Te_3_ nanoplates and carbon nanotubes selected using ultracentrifugation

**DOI:** 10.1038/s41598-023-30175-0

**Published:** 2023-02-21

**Authors:** Tomoyuki Chiba, Hayato Yabuki, Masayuki Takashiri

**Affiliations:** Department of Materials Science, Tokai University, Hiratsuka, Kanagawa 259-1292 Japan

**Keywords:** Carbon nanotubes and fullerenes, Thermoelectric devices and materials

## Abstract

Thermoelectric generators with flexibility and high performance near 300 K have the potential to be employed in self-supporting power supplies for Internet of Things (IoT) devices. Bismuth telluride (Bi_2_Te_3_) exhibits high thermoelectric performance, and single-walled carbon nanotubes (SWCNTs) show excellent flexibility. Therefore, composites of Bi_2_Te_3_ and SWCNTs should exhibit an optimal structure and high performance. In this study, flexible nanocomposite films based on Bi_2_Te_3_ nanoplates and SWCNTs were prepared by drop casting on a flexible sheet, followed by thermal annealing. Bi_2_Te_3_ nanoplates were synthesized using the solvothermal method, and SWCNTs were synthesized using the super-growth method. To improve the thermoelectric properties of the SWCNTs, ultracentrifugation with a surfactant was performed to selectively obtain suitable SWCNTs. This process selects thin and long SWCNTs but does not consider the crystallinity, chirality distribution, and diameters. A film consisting of Bi_2_Te_3_ nanoplates and the thin and long SWCNTs exhibited high electrical conductivity, which was six times higher than that of a film with SWCNTs obtained without ultracentrifugation; this is because the SWCNTs uniformly connected the surrounding nanoplates. The power factor was 6.3 μW/(cm K^2^), revealing that this is one of the best-performing flexible nanocomposite films. The findings of this study can support the application of flexible nanocomposite films in thermoelectric generators to provide self-supporting power supplies for IoT devices.

## Introduction

Thin-film thermoelectric generators (TEGs) are gaining increasing interest as power supplies for sensors and Internet of Things (IoT) devices^1–4^. TEGs produce electrical power from various heat sources, such as the human body, industrial waste heat, and natural heat^5–7^. The power supplies for sensors and IoT devices must possess flexibility and a small size but need not generate high electric power^8^. The requirements of sensors and IoT devices align with the characteristics of thin-film TEGs. The efficiency of a TEG directly depends on the performance of the thermoelectric material, which is expressed as the dimensionless figure of merit (*ZT*), defined as *ZT* = *σS*^2^*T*/*κ*, and the power factor (*PF*), defined as *PF* = *σS*^2^, where *σ*, *S*, and *κ* are the electrical conductivity, Seebeck coefficient, and thermal conductivity, respectively.

Among thermoelectric materials, bismuth telluride (Bi_2_Te_3_) and carbon nanotubes (CNTs) are the primary candidates for the aforementioned applications. Bi_2_Te_3_ was developed in the 1950s and exhibits the highest thermoelectric performance near 300 K^9,10^. Bi_2_Te_3_ has a rhombohedral tetradymite-type crystal structure, with the space group $D_{3d}^{5} (R\mathop 3\limits^{ - } m)$, and its unit cell is described as hexagonal. Owing to this structure, Bi_2_Te_3_ crystals are grown as hexagonal nanoplates in the solution process^11–13^. Bi_2_Te_3_ nanoplates are approximately 1 μm in diameter and tens of nanometers in thickness. This structure contributes to improving the thermoelectric performance owing to the low-dimensional effect and quantum size effect^14–16^. In previous studies, hexagonal Bi_2_Te_3_ nanoplates were synthesized by solvothermal synthesis under various conditions^17–19^, and Bi_2_Te_3_ nanoplate films were prepared using drop-casting^20–23^. Furthermore, CNTs are classified into multi-walled CNTs (MWCNTs), fabricated in 1991, and single-walled CNTs (SWCNTs), fabricated in 1993^24,25^. MWCNTs exhibit metallic characteristics and SWCNTs exhibit metallic or semiconducting characteristics depending on their structure, which is characterized by the chiral index (*n*,*m*)^26^. SWCNTs with semiconducting characteristics have been used as thermoelectric materials^27–31^. The performance of SWCNTs is inferior to that of Bi_2_Te_3_, but SWCNTs have excellent characteristics, including flexibility, heat resistance, and nontoxicity. Therefore, many researchers have attempted to improve the thermoelectric performance of SWCNTs^32–36^.

A favorable approach for improving thermoelectric performance is to fabricate nanocomposites based on Bi_2_Te_3_ nanoplates and semiconducting SWCNTs^37–39^. Jin et al. reported a flexible thermoelectric material composed of highly ordered Bi_2_Te_3_ nanocrystals anchored on an SWCNT network^31^. Hosokawa and Takashiri and Yabuki et al. developed nanocomposite films based on Bi_2_Te_3_ nanoplates and SWCNTs by drop-casting followed by thermal annealing^40,41^. A key factor for increasing the thermoelectric performance of nanocomposites is the quality of SWCNTs. As-synthesized SWCNTs (pristine SWCNTs) exhibit many types of structures with different lengths and chiralities^42,43^. When the optimal SWCNTs with suitable structures are selected, the thermoelectric performance of nanocomposite films based on Bi_2_Te_3_ nanoplates and SWCNTs can be further improved.

In this study, ultracentrifugation is performed for the selection of SWCNTs. Ultracentrifugation is known to select SWCNTs based on their length and chirality in a scalable manner^44–47^. Bi_2_Te_3_ nanoplates are prepared via solvothermal synthesis. The dispersion solution is formed with the selected SWCNTs and Bi_2_Te_3_ nanoplates, and the nanocomposite films are formed on a flexible sheet using the solution by drop-casting, which is a simple and cost-effective wet process. For comparison, pristine SWCNTs are used to form nanocomposite films. The structure and thermoelectric properties of the nanocomposite films are analyzed, and the effectiveness of the selection of SWCNTs is evaluated.

## Experimental procedures

**Figure 1:**
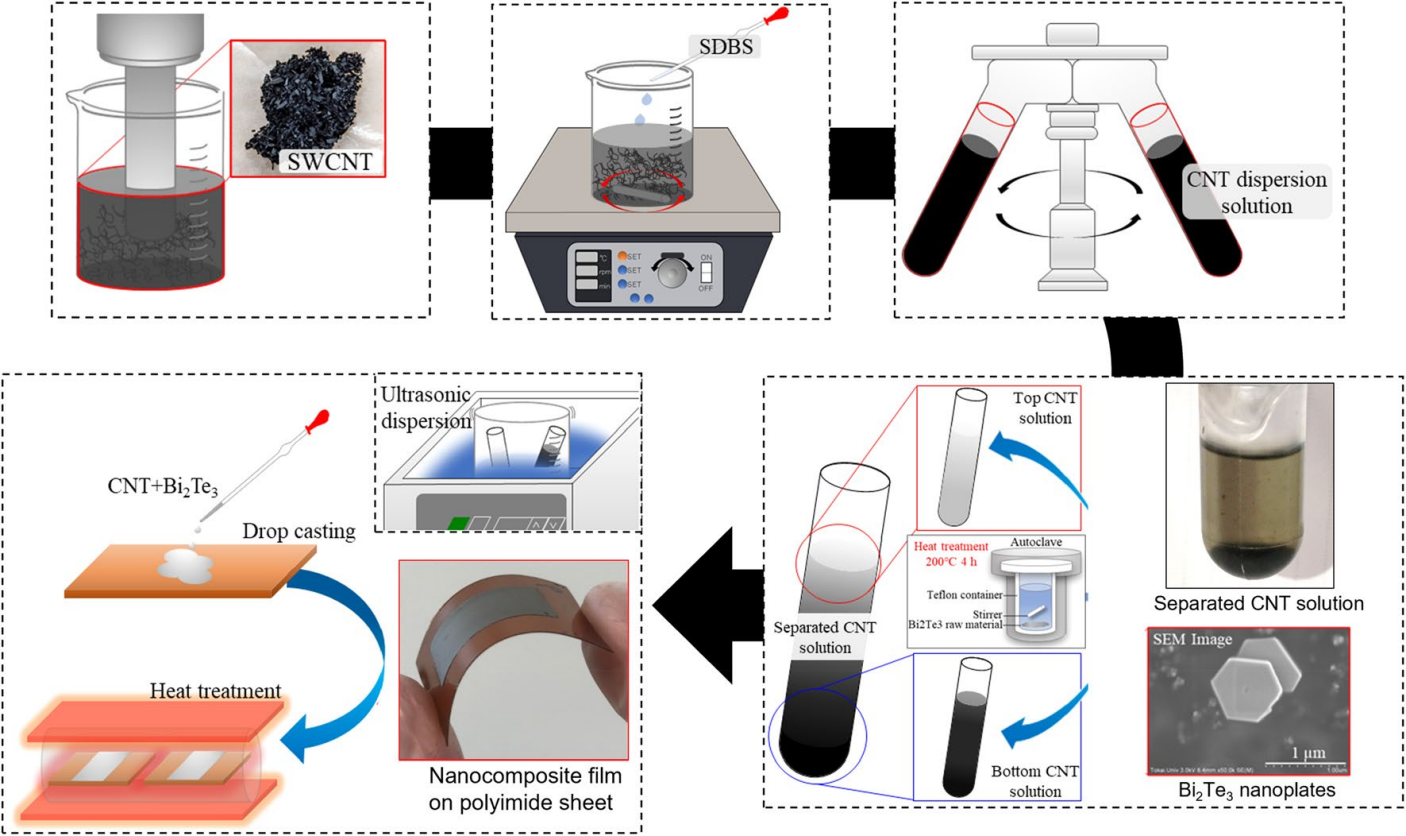
Fabrication process of nanocomposite flexible films based on Bi_2_Te_3_ nanoplates and selected SWCNTs using ultracentrifugation.

A schematic diagram of the fabrication process of flexible nanocomposite films based on Bi_2_Te_3_ nanoplates and SWCNTs is shown in Fig. 1. The Bi_2_Te_3_ nanoplates were synthesized using a solvothermal method. The detailed experimental setup was described in detail in previous reports^21,48^. Briefly, the system comprised a stainless-steel autoclave with a built-in Teflon container, a hot plate with a magnetic stirrer, and heat blocks. The precursor solution and stir bar were placed in an autoclave with an internal volume of 50 cm^3^. The solvents used were analytical-grade Bi_2_O_3_ (purity 99.9%, Fujifilm Wako Co.), TeO_2_ (purity 99.9%, Kojundo Chemical Laboratory), ethylene glycol (purity 99.5%, Fujifilm Wako Co.), polyvinylpyrrolidone (PVP) (purity 99.9%, Fujifilm Wako Co., K30, Ms ~ 40,000), and sodium hydroxide (NaOH) (purity > 97.0%, Fujifilm Wako Chemical Co.) without further purification. Bi_2_Te_3_ nanoplates were fabricated according to the following procedure: 0.4 g of PVP was dissolved in ethylene glycol (18 mL), followed by the addition of Bi_2_O_3_ (20 mM), TeO_2_ (70 mM), and 2 mL of NaOH solution (5.0 M). The resulting precursor solution was then sealed in an autoclave. The autoclave was then heated and maintained at 200 °C for 4 h, with stirring at 500 rpm. After the synthesis, the products were allowed to cool naturally below 70 °C. The products were then collected by centrifugation and washed several times with distilled water and absolute ethanol. Finally, the products were dried under a vacuum at 60 °C for 24 h.

Super-growth carbon nanotubes (SGCNTs) (ZEONANO SG101, purity > 99%, ZEON) were used as SWCNTs. The dispersion solution was prepared by adding 0.5 wt.% of SGCNT powder in 6 mL of ethanol, followed by homogeneously dispersing it using an ultrasonic homogenizer (Emerson SFX25) at 20 W for 45 min. As a surfactant, 2 mL of a sodium dodecylbenzene sulfonate (SDBS) standard solution (Fujifilm Wako Co.) was added to the dispersion solution, followed by stirring at 500 rpm for 30 min using a stirrer (AZ-1 Corporation DP-1L). The dispersion solution (3 mL) was placed in a centrifuge tube, and ultracentrifugation was performed at a rotation speed of 46,000 rpm (average force of 88,000 g) for 1 h. The weight fraction of SWCNTs in the supernatant layer was approximately 10% of the initial amount of SWCNTs used in the solution.

The process conditions for preparing the nanocomposite films were determined based on our previous report^40^. After ultracentrifugation, 1 mL from the top of the solution and 1 mL from the bottom were extracted, and 10 mg of Bi_2_Te_3_ nanoplates were added to each solution. The solution was then drop-casted onto a polyimide substrate using a metal wall. The nanocomposite films were 22 mm long and 12 mm wide, with a thickness of approximately 2 μm. After drying the nanocomposite films in air, they were thermally annealed at 250 °C to evaporate residual solvents in the thin films and to completely connect the Bi_2_Te_3_ nanoplates and SWCNTs. A heating furnace was filled with a mixture of Ar (95%) and H_2_ (5%) at atmospheric pressure. The temperature was maintained at 250 °C for 1 h. Following thermal annealing, the samples were allowed to cool naturally below 70 °C in the furnace. The flexibility of the nanocomposite films was confirmed using the bending test. To evaluate the effect of ultracentrifugation, nanocomposite films composed of Bi_2_Te_3_ nanoplates and SWCNTs were prepared with no ultracentrifugation, that is, with pristine SWCNTs, under the same preparation conditions.

The precise structure of the Bi_2_Te_3_ nanoplates was analyzed using high-resolution transmission electron microscopy (TEM) (JEOL JEM-ARM200F) and selected-area electron diffraction (SAED) at an accelerating voltage of 200 kV. The phase purity and crystal structure of the nanoplates were characterized by X-ray diffraction (XRD) (D8 ADVANCE) using Cu-K*α* radiation (*λ* = 0.154 nm, with 2*θ* ranging from 10° to 80°). The atomic composition of the nanoplates was determined using an electron probe microanalyzer (EPMA, Shimazu, EPMA-1600) and calibrated using the ZAF4 program supplied with the EPMA-1610 device.

The crystallinity and characteristics of the SWCNTs were characterized using Raman spectroscopy with a 532 nm laser source wavelength (XploRA HORIBA). The morphologies of the nanocomposite films of Bi_2_Te_3_ nanoplates and SWCNTs were investigated using field-emission scanning electron microscopy (Hitachi S-4800). The precise morphology and structure of the SWCNTs were analyzed by high-resolution TEM (JEOL JEM-2100F) at an accelerating voltage of 200 kV. The in-plane electrical conductivities (*σ*) of the samples were measured at 300 K using a four-point probe method (Napson RT-70V). The in-plane Seebeck coefficients (*S*) of the samples were measured at 300 K^49–51^. One end of the film was connected to a heat sink, and the other end was connected to a heater. Two 0.1-mm-diameter K-type thermocouples, placed 13 mm apart, were pressed near the center of the thin films. The temperature difference between the thermocouples was varied from 1 to 4 K, and the thermoelectric voltage was recorded at intervals of 1 K. The Seebeck coefficient was estimated according to the V-K slope using a linear approximation. The in-plane power factor (*σS*^2^) was obtained from the experimentally measured electrical conductivity and the Seebeck coefficient.

## Results and discussion

### Structural properties of Bi_2_Te_3_ nanoplates

**Figure 2:**
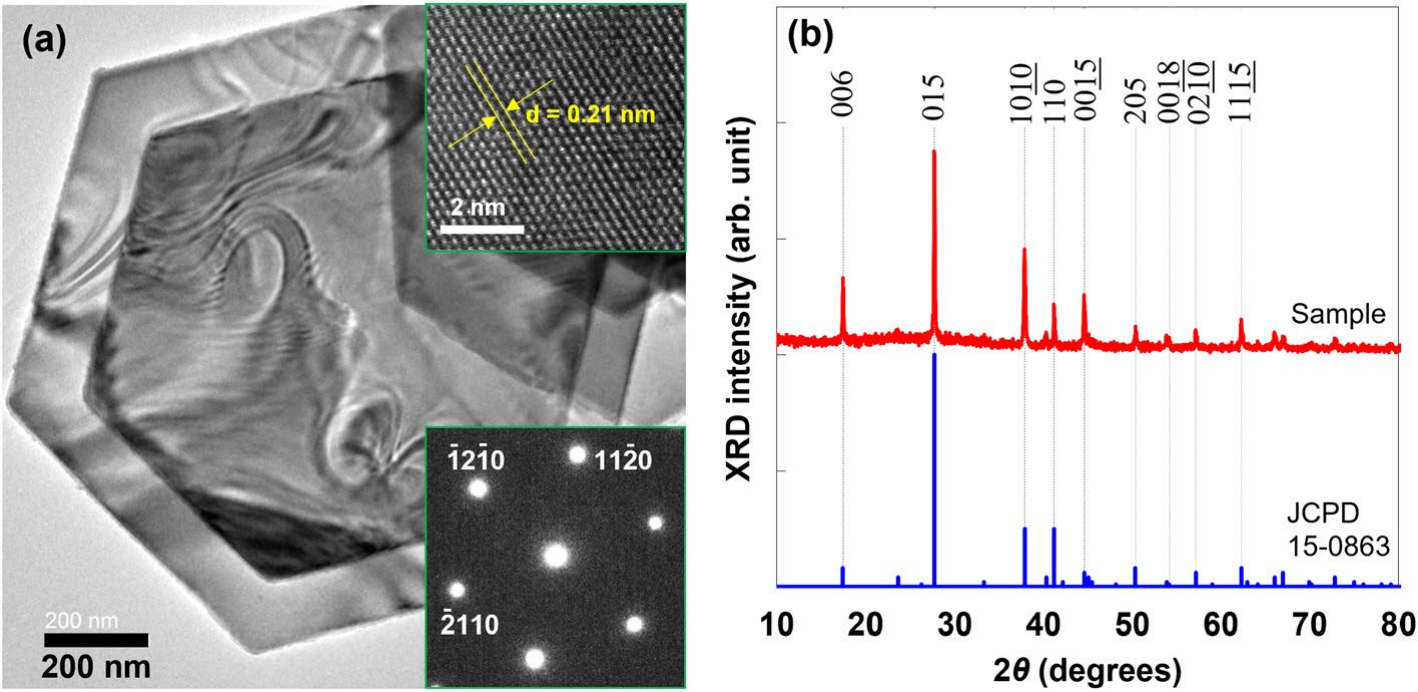
(**a**) TEM image of Bi_2_Te_3_ nanoplates prepared by solvothermal synthesis. The insets show the HRTEM image and SAED pattern. (**b**) X-ray diffraction patterns of the Bi_2_Te_3_ nanoplates.

A TEM image of a typical Bi_2_Te_3_ nanoplate is shown in Fig. 2a. The Bi_2_Te_3_ nanoplates exhibited a regular hexagonal shape with an approximate lateral size of 1–2 μm. The nanoplates were sufficiently thin (less than 50 nm) that the overlap of the nanoplates could be observed. The SAED pattern shown in the inset of Fig. 2a was indexed to the [00*l*] zone axis of rhombohedral Bi_2_Te_3_, indicating that the nanoplate was single crystalline. The high-resolution TEM (HRTEM) image in the inset of Fig. 2a shows that the lattice fringes were also structurally uniform, with a spacing of 0.21 nm, which is in good agreement with the *d* value of the (110) planes of rhombohedral Bi_2_Te_3_. The phase purity and crystal structure of the Bi_2_Te_3_ nanoplates were examined using XRD analysis, as shown in Fig. 2b. The peaks observed in the XRD patterns of the nanoplates were indexed to the standard diffraction pattern of Bi_2_Te_3_ (JCPDS 15-0863). The main peaks were observed in the *c*-axis-oriented (00*l*), (015), and (1010) planes. The atomic ratio of nanoplates (Te/(Bi + Te)) determined using EPMA analysis was 60.6 at.%, which was very close to the stoichiometric proportion of 60.0 at.%. In addition, oxygen was detected in the EPMA analysis, indicating either adsorption of oxygen molecules on the nanoplate surface or formation of natural oxide layers.

### Structural properties of SWCNTs

**Figure 3:**
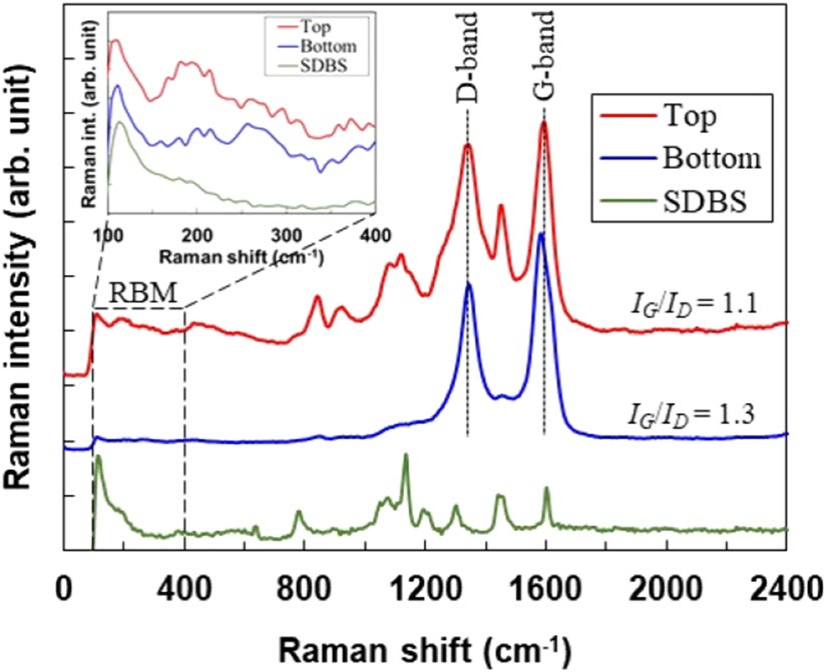
Raman spectra of SWCNTs in the top and bottom solutions. The inset shows a detailed analysis of the RBM modes ranging from 100 to 400 cm^-1^.

The Raman spectra of the SWCNTs in the top and bottom solutions are shown in Fig. 3. For comparison, the Raman spectra of the SDBS are shown in the figure. The Raman spectra of the SWCNTs in the top solution included the peaks of the SWCNTs and SDBS, whereas those of the SWCNTs in the bottom solution only exhibited the peaks of the SWCNTs, even though the cleaning process of the SWCNTs after ultracentrifugation was the same for the top and bottom solutions. This indicates that the molecules of SDBS were more firmly attached to the SWCNT surface in the top solution. The intensity ratio of the G and D bands, *I*_*G*_*/I*_*D*_, which shows the crystallinity of SWCNTs, was 1.1 and 1.3 for the SWCNTs in the top and bottom solutions, respectively. Therefore, the crystallinity of the SWCNTs did not vary significantly between the top and bottom solutions. In the inset of Fig. 3, the SWCNTs in the top and bottom solutions exhibited several radial breathing mode (RBM) peaks in the range of 100–400 cm^−1^, indicating that SWCNTs with different chirality distributions and diameters existed. Consequently, the crystallinity, chirality distribution, and diameters of the SWCNTs were not selected by ultracentrifugation.

### Structural and thermoelectric properties of nanocomposite films

**Figure 4:**
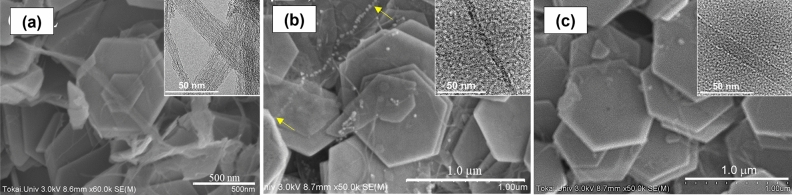
SEM image of nanocomposite films based on Bi_2_Te_3_ nanoplates and different SWCNTs. The insets show the HRTEM images of each SWCNT. (**a**) Pristine SWCNTs, (**b**) SWCNTs in the top solution, and (**c**) SWCNTs in the bottom solution.

Figure 4 shows SEM images of the surface morphologies of the nanocomposite films with Bi_2_Te_3_ nanoplates and SWCNTs obtained from different positions in the aforementioned solution. For comparison, the SEM image of the nanocomposite film with pristine SWCNTs is shown in Fig. 4a. Regular hexagonal Bi_2_Te_3_ nanoplates, with an average diameter of approximately 1 μm, were relatively well-aligned. There were variations in the diameter of the SWCNT bundles, and the maximum diameter observed was approximately 100 nm. The SWCNT bundles were observed by HRTEM, as shown in the inset of Fig. 4a. Owing to the uneven distribution of the SWCNT bundles, the positions at which nanoplates could be connected were limited. Upon using the SWCNTs in the top solution (Fig. 4b), the Bi_2_Te_3_ nanoplates were relatively well-aligned to the in-plane direction. The diameters of the SWCNT bundles were significantly smaller than those of the pristine SWCNT bundles, as shown in Fig. 4a. This indicates that the SWCNT bundles were unraveled by ultracentrifugation, which is revealed by the HRTEM image in the inset of Fig. 4b. Many thin and long SWCNTs were uniformly attached to the Bi_2_Te_3_ nanoplates. As the length of the SWCNTs was longer than that of the nanoplates, the SWCNT connected the surrounding nanoplates. The location of yellow arrows in the figure shows the most typical SWCNTs. When the SWCNTs in the bottom solution were used (Fig. 4c), thin SWCNTs were observed on the surface of the nanoplates, and the SWCNT connected the surrounding nanoplates. The diameter of the SWCNTs in the bottom solution was comparable to that of the SWCNTs in the top solution, which was observed by the HRTEM image shown in the inset of Fig. 4c.

The thermoelectric properties of the nanocomposite films are presented in Table 1. The electrical conductivity of the film with SWCNTs from the top solution was 370 S/cm, which was approximately six times higher than that of the film with SWCNTs from the bottom solution. The clear mechanism could not be identified. However, a possible explanation is that the SWCNTs in the top solution were firmly coated with the residual surfactant, as shown in Fig. 3, and that the residual surfactant contributed to a strong connection between the SWCNTs and nanoplates^52^. Consequently, sodium ions in the SDBS surfactants were considered to increase the electrical conductivity. The detailed analysis of the mechanism should be performed using a molecular dynamics simulation in the future. The electrical conductivity of the film with pristine SWCNTs was also low, possibly because the current path was restricted owing to the non-uniform distribution of the SWCNT bundles^41^. The Seebeck coefficient of the film with SWCNTs from the top solution was exhibited at n-type characteristic and a value of − 131 μV/K, which was approximately 10% higher than those of the films with SWCNTs from the bottom solution and pristine SWCNTs. The phenomena occurred because of the decreasing contact resistance between the SWCNTs and nanoplates. The power factor of the film with SWCNTs from the top solution was 6.3 μW/(cm K^2^), which was approximately eight times higher than that of the films with SWCNTs from the bottom solution and pristine SWCNTs, mainly because of the significant increase in electrical conductivity. As a result, by performing ultracentrifugation with the SDBS surfactant to select the SWCNTs, the power factor can be greatly improved. The thermoelectric properties of flexible nanocomposite films were compared to those of flexible nanocomposite films reported in the literature. The power factor of the nanocomposite films of Bi_2_Te_3_ nanoplates/SWCNTs (top) was higher than that of the nanocomposite film with SWCNTs using similar wet processes and with reduced graphene oxide nanosheets (rGO)^53–55^. However, the top value in this study was lower than the value (18.4 μW/(cm⋅K^2^) of flexible nanocomposite film using a sputtering (dry process), possibly because highly oriented nanoplates were obtained^31^. Therefore, the selection of SWCNTs using ultracentrifugation with a surfactant improved the thermoelectric performance via a simple and cost-effective method. To further enhance the performance, an effective way is to improve the orientation of the nanoplates by optimizing the process.

**Table 1:** Thermoelectric properties of nanocomposite flexible films.

Nanocomposite flexible films	Film fabrication	σ [S/cm]	*S* [μV/K]	*P.F.* [μW/(cm K^2^)]	Ref.
Bi_2_Te_3_ nanoplates/SWCNTs (top)	Wet	370	− 131	6.3	This work
Bi_2_Te_3_ nanoplates/SWCNTs (bottom)	Wet	61	− 118	0.8	This work
Bi_2_Te_3_ nanoplates/SWCNTs (pristine)	Wet	56	− 119	0.8	^41^
Bi_2_Te_3_ nanoplates/SWCNTs	Wet	510	− 100	5.1	^53^
Bi_2_Te_3_ nanoplates/SWCNTs	Wet	152	− 37	0.2	^54^
Bi_2_Te_3_ nanoplates/rGO	Wet	200	− 128	3.3	^55^
Bi_2_Te_3_ nanoplates (Scaffold)/SWCNTs	Dry	940	− 140	18.4	^31^

## Conclusion

To improve the thermoelectric performance of nanocomposite films based on Bi_2_Te_3_ nanoplates and SWCNTs, ultracentrifugation was used to select suitable SWCNTs. After ultracentrifugation, the dispersion solution was divided into two parts, the top and the bottom, and each part was dried. The SWCNTs from each part were mixed with Bi_2_Te_3_ nanoplates in ethanol and flexible nanocomposite films were prepared on a polyimide sheet by drop casting. The nanocomposite film fabricated with SWCNTs from the top solution exhibited a higher electrical conductivity than that of the nanocomposite films with SWCNTs from the bottom solution and SWCNTs without ultracentrifugation. This phenomenon occurred possibly because the residual surfactant contributed to a strong connection between the SWCNTs and nanoplates. In future work, several other approaches will be further investigated to deduce the mechanism. The power factor was 6.3 μW/(cm K^2^), revealing that this was one of the best-performing flexible nanocomposite films. Therefore, this study demonstrated an improvement in the thermoelectric performance of nanocomposite films by the selection of SWCNTs using ultracentrifugation with a surfactant. The findings of this study can support the application of nanocomposite films in TEGs for providing self-supporting power supplies for IoT devices. Further research should focus on increasing the electrical conductivity while maintaining a high Seebeck coefficient.

## Data Availability

The datasets used and/or analyzed during the current study available from the corresponding author on reasonable request.
